# Research on the community electric carbon emission prediction considering the dynamic emission coefficient of power system

**DOI:** 10.1038/s41598-023-31022-y

**Published:** 2023-04-05

**Authors:** Hui Yu, Yang Yang, Bin Li, Bowen Liu, Yuanhu Guo, Yunqi Wang, Zhongfu Guo, Ronghua Meng

**Affiliations:** 1 State Grid Beijing Urban District Power Supply Company, Beijing, 100032 China; 2 Hubei Key Laboratory of Construction and Management in Hydropower Engineering, China Three Gorges University, Yichang, 443002 Hubei China; 3 Intelligent Manufacturing Innovation Technology Center, China Three Gorges University, Yichang, 443002 Hubei China

**Keywords:** Electrical and electronic engineering, Energy grids and networks

## Abstract

Based on the counted power system emission factors of North China Power Grid, a community carbon emissions sample database is constructed. The support vector regression (SVR) model is trained to forecast the power carbon emissions, which is optimized by genetic algorithm (GA). A community carbon emission warning system is designed according the results. The dynamic emission coefficient curve of the power system is obtained by fitting the annual carbon emission coefficients. The time series SVR carbon emission prediction model is constructed, while the GA is improved to optimize its parameters. Taking Beijing Caochang Community as an example, a carbon emission sample database is generated based on the electricity consumption and emission coefficient curve to train and test the SVR model. The results show that the GA–SVR model fits well with the training set and the testing set, and the prediction accuracy of the testing set reaches 86%. In view of the training model in this paper, the carbon emission trend of community electricity consumption in the next month is predicted. The carbon emission warning system of the community is designed, and the specific strategy of community carbon emission reduction is proposed.

## Introduction

In the background of prominent global climate issues and increasing heat island effect, low-carbon development has become the requirement of the times. With the acceleration of China urbanization process, the urban residents continue to expand. Industrial clusters have accelerated development, and energy consumption and carbon emissions have been increasing continuously. The problem of high carbon emissions in cities has become particularly important. The community, especially the high-density community, is the main unit of the city. With the implementation of policies such as coal to electricity, the electricity carbon emissions become increasingly the main community carbon emissions. Prediction of the community electricity carbon emissions shows great significance to promote low-carbon life of residents and build low-carbon cities.

In recent years, as the largest developing country, China has a large economy and a high proportion of population. China consumes a lot of fossil fuels and its carbon emissions are growing rapidly. The total carbon emissions increased continuously after the total carbon emissions of China exceeded the United States firstly in 2008. China has become the largest carbon emitter since its total carbon emissions increased doubled the United States in 2019. To solve the problem of global warming and reduce greenhouse gas emissions, low carbon development has become the consensus of the world. In 2014, China first proposed the 2030 carbon peak plan in the *U.S.–China Joint Declaration on Climate Change*, and announced China's carbon peak and carbon neutrality goals in 2020. At the same time, in January 2022, the State Council issued the *14th Five-Year Plan for Energy Conservation and Emission Reduction Comprehensive Work Plan*, which improved policy mechanisms, deployed key projects, and clarified the near-term emission reduction goals of China.

In order to serve the national goal of carbon emission reduction, carbon emission prediction models and solutions have become the focus of research by scholars at home and abroad. At present, there are more forecasts^1–3^ for carbon emissions within a certain industry. In the power industry, carbon emissions are usually forecasted from economic and power consumption aspects. Researchers have carried out a series of studies based on the influencing factors of carbon emissions^4,5^, regression models^6,7^, system dynamics models^8–10^ and nonlinear models^11,12^. For example, in terms of influencing factors, He et al.^13^ pointed out that carbon emissions from the power sector account for more than 40% of the total emissions in China. They examined the influencing factors of carbon emissions from the power sector in China at both national and provincial levels, with economic growth being the main driver and power consumption intensity, thermal power generation energy intensity and power mix being the main inhibiting factors. Sun Wei et al.^14^ used stochastic frontier analysis to screen factors affecting carbon emission intensity from the perspective of carbon emission efficiency, and constructed a carbon emission intensity prediction model based on factor analysis and extreme learning machine. McKibbin et al.^15^ adopted an approach developed using the G-Cubed multi-country model in which economic structure and emission outcomes were determined simultaneously. The framework for emission forecast should focus on the time-variant sources of economic growth and structure of the global economy.

The construction of prediction model is also the focus of research on carbon emissions: based on linear fitting prediction, mainly including IPAT equation, carbon emission environment Kuznets curve, grey prediction, and other models. In the early 1970s, Ehrlich et al.^16^ proposed the IPAT equation, which used mathematical equations to quantify the relationship between economic growth and resource environment. This paper applied IPAT equation to analyze the factors affecting carbon emissions and to predict carbon emissions. By 1990s, Grossman et al.^17^ found that there was an inverted U type relationship between economic growth and environmental quality. They proposed the carbon emission environmental Kuznets curve, which has received much attention from scholars. However, the application of the method by researchers shows that, it is currently difficult to explain the contradictory conclusions generated by the study, which reflects the complex relationship between carbon emissions and the economy; on the other hand, the defects of the environmental Kuznets curve theory itself cannot be ignored^18^. For the complex relationship between various factors in power systems, methods to deal with multi-factor problems have been proposed one after another. Grey prediction models based on grey system theory^19^ are widely used in several fields. Several scholars^20–22^ have used grey prediction models to forecast carbon emissions. The grey prediction model is widely used, easy to calculate, less data sample requirement, higher accuracy, and suitable for short- and medium-term forecast. For long-term forecast, the uncertainty of future perturbation factors will make the forecast accuracy lower. Xiong et al.^23^ developed a new multivariate grey model based on linear time-varying discrete parameters. In this model, a linear time-varying function was introduced into the traditional model to dynamically optimize the fixed parameters that can only be used for static analysis. This method does not change the essence of linear prediction, although it improves some prediction accuracy.

In recent years, with the development of big data technology, machine learning methods began to be widely used in load prediction and state estimation, and achieved good application results, so some scholars began to use machine learning^24–26^ for carbon emission prediction. Support vector regression (SVR) is also widely used in time series prediction problems, and it performs superior generalization ability and can better avoid local optima through parameter optimization. Wang et al.^27^ proposed an online SVR model to predict air pollutant levels in forward time series. Melahat et al.^26^ used deep learning (DL), support vector machine (SVM) and artificial neural network (ANN) algorithms to predict carbon emissions in the electricity production sector in Turkey, respectively. Li et al.^28^ used genetic algorithm (GA) to optimize the weights and thresholds of the SVR to predict CO_2_ emissions in Beijing from 2016 to 2020 by scenario analysis. Saleh et al.^29^ established an SVR model with energy consumption such as electric energy and coal combustion as input variables, which directly affect the increase of CO_2_ emissions. The SVRs have been used successfully in prediction of time series problems in many ways.

Although a series of studies have been conducted by scholars on the carbon emissions prediction, few articles have been published on the carbon emissions prediction for community electricity. Usually, the carbon emissions of community electricity consumption are mainly related to the community electricity consumption and the carbon emission factor of electricity supply. Carbon emission coefficient^30^ is the number of carbon emission mass per unit of energy produced during the combustion or use of each energy source. In the existing literature, most of them use fixed carbon emission factors, or guideline factors issued by the state. In fact, the carbon emission factor of electric energy varies dynamically depending on the energy ration of power production by the power supply company. Especially for community, city, and national electricity carbon emission forecast, the small changes of carbon emission coefficients have a great impact on the total carbon emission due to the large consumption of electric energy. The traditional forecast methods have some problems such as unstable regression and difficult to determine the influencing factors, which will reduce the scientific accuracy of forecast. After research, it is found that the changes of carbon emissions and power supply carbon emission coefficients in a certain region are time-series correlated, so the historical data of carbon emissions can be used to predict the future carbon emissions.

Now, China has proposed the concept of smart community. The community will fully implement the construction requirements of new electric power system, complete electric energy substitution, replace coal with electricity and gas with electricity. The electric energy becomes the main energy source of the community. To carry out the promotion of terminal electric energy substitution products, residents' clean energy and reliable electricity use will be effectively guaranteed. The well-being of regional people's livelihood will be improved in an orderly manner. Therefore, the prediction of carbon emission for community electric energy can effectively represent the overall carbon emission of the community, and is increasingly meaningful for community carbon emission reduction.

Based on the complex nonlinear carbon emission prediction system, this paper intends to calculate the annual carbon emission coefficient according to the power production of North China Power Grid (NCPG). The dynamic carbon emission factor curve of electricity will be fitted. Considering both the dynamic carbon emission coefficient on the supply side and the electricity consumption on the demand side, a sample database of carbon emissions in the community will be built. The GA optimization SVR model (GA–SVR) is improved and designed to predict the carbon emissions of community electricity consumption.

## Carbon emission sample database

Different power supply networks have different ratios of energy generation; thus, the same carbon emission factor is inappropriate. Even if the energy ratio of the same power supply network is different in different years, the carbon emission factor is dynamic. This paper collects the energy consumption of NCPG from 2011 to 2020, calculates the annual carbon emission factors, and fits the carbon emission factors after 2021.

### Statistical of annual power system emission coefficient

According to Power system emission coefficient calculation tool^31^, the calculation method of carbon dioxide baseline emission factor OM in China power grid. Based on the total net power generations, fuel types and fuel consumptions of all power plants in the power system, the formula (1) is as follows:

1 $$ EF_{grid,OMsimple,y} = \frac{{\sum\nolimits_{i} {\left( {FC_{i,y} \times NCV_{i,y} \times EF_{{CO_{2} ,i,y}} } \right)} }}{{FC_{y} }} $$

$EF_{grid,OMsimple,y}$ is the simple electricity marginal emission factor *OM* (*tCO*_2_/MWh) of the power system when the research object in Year *y*. *EG*_*y*_ is the net total generation of the power system in Year *y*. That is, the total electricity (MWh) supplied to the grid by all units other than the must operating cost. *FC*_*i*,*y*_ is the total fuel consumption (mass or volume unit) of the above units in Year *y*. *NCV*_*i*,*y*_ is the average low calorific value (*GJ*/mass or volume unit), while the $EF_{{CO_{2} ,i,y}}$ is the *CO*_2_ emission factor of fuel i in Year *y*, (*tCO*_2_/*GJ*).Then, *i* is the type of fossil fuel consumed by the power system in year y, while y is the year that the data are available for project submission. In the case of power exchange between power grids, the power exchange between power grids will produce net receiver party. The simple electricity marginal emission factor is equal to the weighted average value of the unit electricity emission factor of the local power plant and the unit electricity receivers. The emission factor of unit electricity of local power plants is calculated according to the above formula, and the net receive electricity adopts the simple marginal emission factor of transferred power grid.

The data of power generation, fuel consumption for power generation and low calorific value of power generation fuel for OM are originated respectively from *China Energy Statistical Yearbook*^32^. The data of auxiliary power consumption rate are derived from *China Electric Power Yearbook*. The data of power exchange between power grids are from *Compilation of electric power industry statistics*. The CO_2_ emission factors of fuel are derived from Table 1.4 in Chapter I of the *Guidelines for the preparation of the 2006 IPCC national inventory* Energy Volume. The lower limit of 95% confidence interval of each fuel emission factor is determined according to the principle of conservatism.

### Establishment of dynamic carbon emission coefficient regression model

According to the principle of least squares, the fitting curve objective is constructed as formula (2):

2 $$ \mathop {\min }\limits_{\varphi } \sum\limits_{i = 1}^{n} {\delta_{{\text{i}}}^{2} } = \sum\limits_{i = 1}^{n} {\left( {\varphi \left( {x_{i} } \right) - y_{i} } \right)^{2} } $$

where *φ*(*x*_*i*_) represents the ordinate of the fitting model function, while *y*_*i*_ expresses the ordinate of the real scatter point. The smallest interpolation quadratic sum of the ordinates from the function model and scattered points will be considered as the best fit degree.

The polynomial fitting model is set as formula (3):

3 $$ y = a_{0} + a_{1} x + \cdots + a_{k} x^{k} $$

The carbon emission curve is obtained by fitting the calculated annual carbon emission coefficient. Then the daily carbon emission coefficient will appear.

### Calculation of carbon emission

According to the electricity consumption and carbon emission coefficient curve of the community, daily carbon emission can be obtained from the following formula (4).

4 $$ CE = y_{OM} \times E $$

where *CE* is the carbon emission, *y*_*OM*_ is the carbon emission coefficient curve, and *E* is the community electricity consumption.

## Methods

Due to the continuous construction of communities and changes in resident living habits, the sample data beyond a certain period for carbon emission forecast will inevitably lead to inaccurate prediction. The training sample size is insufficient to support the learning process of prediction model, when the recent sample data are considered merely. The SVR^33^ introduced in this paper is a novel small-sample learning method that can solve the small training sample problems. Due to its wide range of weight parameters, poor training results will appear with empirical parameters, while it will be time-consuming by iterative methods.

In this paper, GA is introduced to synchronously optimize the penalty factor *C* and radial basis function parameter *g* in SVR to obtain the global optimal solution. Compared with traditional optimization methods, GA^34^, based on biological evolution, is a modern intelligent algorithm that simulates the fittest survival in the biological world and the natural genetic mechanism, which has a strong global search ability. Its calculation speed is fast, with high robustness and strong expansibility. The GA is easy to combine with SVR model, which speeds up the solution process of this problem.

### Basic principles of genetic algorithms

GA is stochastic global search and optimization methods developed to mimic the mechanisms of biological evolution in nature. The model simulates the natural selection and genetic mechanism of Darwinian biological evolution process. To find the optimal solution by following the law of survival of the fittest. Keep the excellent individuals and eliminate the bad individuals during the optimization process. The GA optimizes the penalty parameter *C* and kernel function parameter *g* of the SVR model to improve its prediction accuracy, aiming at minimizing the root mean square error (RMSE) of the predicted value and the actual value of the sample. The fitness function formula is:

5 $$ fit = \sqrt {\frac{1}{N}\left( {T_{sim} - T_{train} } \right)^{2} } $$

where *T*_*sim*_ represents the predicted value of the training set, *T*_*train*_ means the actual value of the sample, and N represents the number of samples in the training set.

### Basic principle of SVR

**Figure 1:**
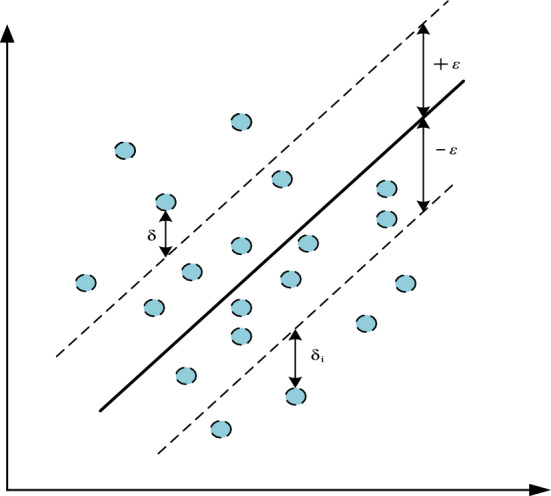
Schematic diagram of support vector product regression.

The SVR is a supervised learning algorithm, which is widely used in discrete prediction and other fields. For a given data set $\{ x_{i} ,y_{i} \}_{i = 1}^{N}$ , N is the sample number in the training set; *x*_*i*_ and *y*_*i*_ are input and output variables respectively. SVR maps the original space of the input data to a higher dimensional feature space through the nonlinear Gaussian kernel function. In the feature space, the problem is transformed into the construction of the optimal linear plane *f*(*x*) = *w*·*φ*(*x*) + *b* for fitting data. Where *b* is offset and *w* is weight. Therefore, by minimizing the vector norm *w*^2^ to find the smoothest function *f*(*x*), the maximum allowable error of the predicted value of each training data is *ε*, as Fig. 1.Then the Lagrange multiplier is used to rephrase the constrained optimization problem as a dual problem. For each constraint, the quadratic programming is used to determine, and then the offset of the optimal weight is calculated to obtain the predicted value. In this way, the previous regression problem can be replaced by a constrained optimization problem. The formulae of objective and constraints are as follows:

6 $$ \min \frac{1}{2}\left\| w \right\|^{2} + C\sum\limits_{i = 1}^{N} {\left( {\delta_{i} + \delta_{i}^{*} } \right)} $$

7 $$ \left\{ {\begin{array}{*{20}l} {\begin{array}{*{20}l} {y_{i} - w \cdot \varphi \left( x \right) - b \le \varepsilon + \delta_{{\text{i}}} } \hfill \\ {w \cdot \varphi \left( x \right) + b - y_{i} \le \varepsilon + \delta_{{\text{i}}}^{*} } \hfill \\ {\updelta _{{\text{i}}} \ge 0} \hfill \\ {\updelta _{{\text{i}}}^{*} \ge 0} \hfill \\ \end{array} ,} \hfill & {{\text{i}} = 1,2, \ldots N} \hfill \\ \end{array} } \right. $$

In mathematic formulae (6) and (7), where *C* is the penalty coefficient, δ_i_ and δ_i_^*^ are both relaxation variables, and *ε* is the loss function. In addition, this model is converted into a pairwise function by multiplier solving method with the Lagrange function. The nonlinear function can be expressed as formula (8):

8 $$ f\left( x \right) = \sum\limits_{i = 1}^{N} {\left( {\alpha_{i} - \alpha_{i}^{*} } \right)K\left( {x_{i} ,x_{j} } \right) + b} $$

where *αi* and $\alpha_{i}^{*}$ denote Lagrange multipliers and *K*(*x*_*i*_, *x*_*j*_) denotes the kernel function. The SVR will have different learning and generalization performance by different kernel functions. The linear kernel function, polynomial kernel function and radial basis function (RBF) are commonly used by SVR. RBF performs better than linear kernels on dealing with nonlinear problems and has fewer hyperparameters than polynomial kernels. Therefore, RBF is widely considered to be an ideal function on handling complex and multidimensional samples. In this paper, RBF is chosen as the kernel function, as formula (9).

9 $$ K\left( {x_{i} ,x_{j} } \right) = \exp \left( { - \frac{{\left\| {x_{i} - x_{j} } \right\|^{2} }}{{2\sigma^{2} }}} \right) $$

where *σ* denotes the bandwidth, which is sufficient to handle a limited number of samples.

### Algorithm implementation

The improved GA–SVR algorithm is implemented as follows:

*Step 1* the obtained carbon emission data are normalized, and the SVR model kernel function RBF and parameters are selected.

*Step 2* parameters *C* and *g* of the SVR model are considered as variables, and optimized through the improved GA.

*Step 3* train and test the SVR model as the decode process of GA.

*Step 4* calculate the fitness function of the GA and save the optimal solution. Judge whether the segment satisfies the stopping condition. If the condition is not satisfied, continue the GA operation to Step 2, while the condition is satisfied, Step 5 will be executed.

*Step 5* forecast 1 month carbon emission values based on known annual carbon emission data.

**Figure 2:**
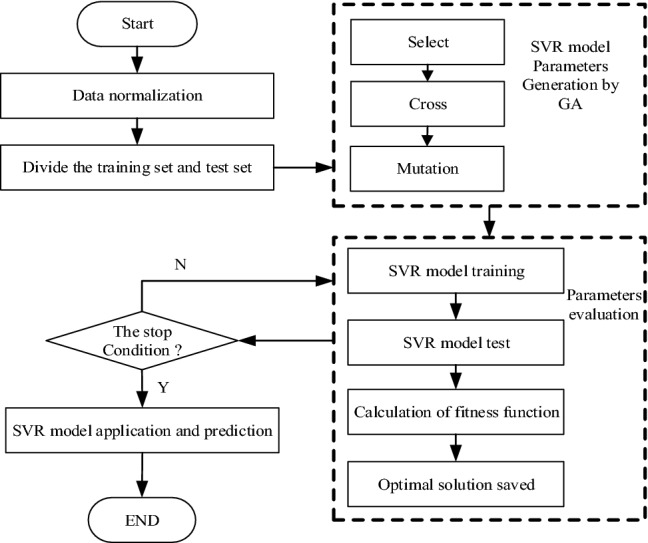
Flow chart of GA–SVR algorithm.

The flow chart of GA–SVR is described in Fig. 2.

## Results

Caochang Community is in Dongcheng District, Beijing, China. It is from Qianmen-East Street in the west to Caochang-shitiao in the east, and starts Xi-damochang Street in the north to Liangguang Street in the south, which covers an area of 586,000 square meters. The population is 9000, including 4817 resident customers and 359 non-residents. In 2021, the power consumption of this area is 14.772 million kwh, and the load of residential customers is account for about 68%. Caochang area is evaluated as A+, which belongs to Tong'an and Anhangxing Districts. The total length of the transmission line is 18.1 km, and the cabling rate achieves 100%. It has 47 shutters and 87 box transformers. The coverage rate of line automation reaches 100%, while the coverage of equipment automation ascends to 92.73%.

The north has higher carbon emissions than the south in China, especially to warm by heating in winter. Large coal consumption leads to high carbon emissions. Caochang Community realizes the electric energy substitution in the city firstly, which becomes the first demonstration block to replace coal and gas with electricity. Since 2007, the region has completed the ‘coal to electricity’, ‘gas to electricity’ work successively. The proportion of regional electric energy in terminal energy consumption is increasing. Since 2018, it has completed the construction of regional hutong overhead lines before others and promoted the three modernizations of power equipment boxes. All these measures are devoted to build a green, low-carbon, clean and livable model of old urban areas. After 2019, 52 Symbiosis Institutes have been connected to power, and have comprehensively promoted terminal power alternative products. The clean and reliable of resident electricity consumption are effectively guaranteed. The improvement of regional resident livelihood is promoted in an orderly manner. So, the carbon emission prediction of electric energy can effectively represent the overall carbon emission of the community.

Taking Caochang Community in Beijing as an example, the carbon emission sample database is generated based on the electricity consumption and emission factor curve for GA–SVR model’s training and testing.

### Dynamic carbon emission coefficient curve regression fitting

**Figure 3:**
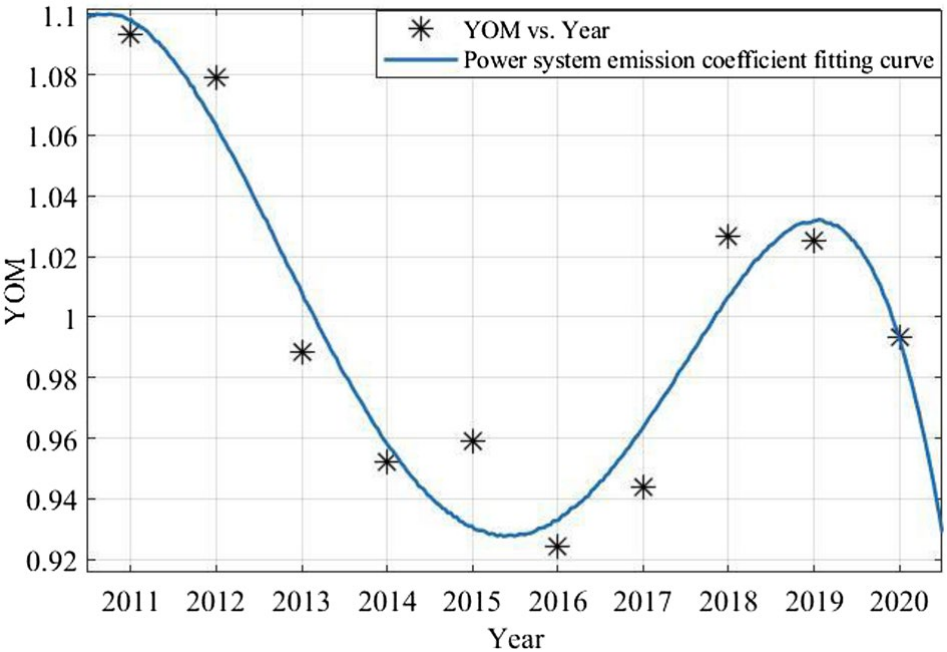
Power system emission coefficient fitting curve.

According to the statistical method of this paper, the annual emission factor of the power system is calculated. The linear fitting is performed by Matlab to obtain the curve shown in Fig. 3.

The curve formula of carbon emission factor obtained through linear fitting is as formula (13):

10 $$ \begin{aligned} y_{OM} & = - 1.547e - 05x^{5} + 0.1554x^{4} - 624.3x^{3} + 1.254e + 06x^{2} \\ & \quad - 1.26e + 09x + 5.063e + 11 \\ \end{aligned} $$

The square root of the error R^2^ = 0.9172, which is greater than 0.9, performs an excellent fitting effect.

From the fitting curve of the annual emission coefficient of the power system, in the process of continuous fluctuation, the coefficient declined steadily. Emission factors from 2011 to 2012 show a high situation. Since 2013, the annual carbon emission factors turn to a significant downward trend. However, during 2018 and 2019, the emission factors increased significantly due to the use of blast furnace gas and converter gas with higher fuel emission factor. The above raw materials are by-products of the production process of steel enterprises. Although the gas improves the carbon emission factor of electric energy during power generation, it forms an industrial ecological chain with the steel metallurgical industry, which can improve the added value of gas.

### Introduction of evaluation metrics

In order to evaluate the prediction effect of GA–SVR model, four evaluation metrics are introduced as follows. Root means square error (RMSE), mean absolute error (MAE), mean bias error (MBE) and R-Square (R^2^). RMSE is used to measure the deviation between the observed value and the true value. MAE and MBE can better reflect the prediction error. R^2^ can reflect the fitting ability from a statistical point of view. The formulae of these evaluation metrics are as follows:

11 $$ RMSE = \sqrt {\frac{1}{N}\sum\limits_{m = 1}^{N} {\left( {y_{m} - \hat{y}_{m} } \right)^{2} } } $$

12 $$ MAE = \frac{1}{N}\sum\limits_{m = 1}^{N} {\left| {y_{m} - \hat{y}_{m} } \right|} $$

13 $$ MBE = \frac{1}{N}\sum\limits_{m = 1}^{N} {\left( {y_{m} - \hat{y}_{m} } \right)} $$

14 $$ R^{2} = 1 - \frac{{\sum\nolimits_{m = 1}^{N} {\left( {y_{m} - \hat{y}_{m} } \right)^{2} } }}{{\sum\nolimits_{m = 1}^{N} {\left( {y_{m} - \overline{y}} \right)^{2} } }} $$

### Prediction result analysis of GA–SVR model

**Figure 4:**
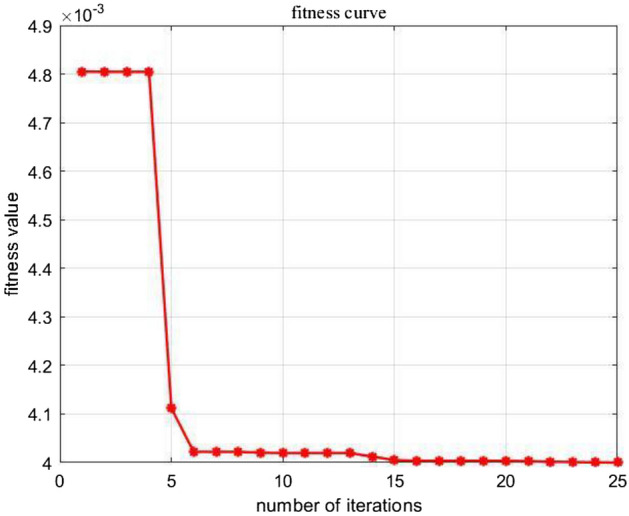
Fitness iteration curve.

In order to analyze the operation effect of the algorithm and the fairness of multi-algorithm comparison, the following algorithms are all implemented by Matlab2020(a). The computer system is Windows 10 with Intel (R) Core (TM) i7-10700 CPU @ 2.90 GHz configuration. Set the parameter values and ranges for GA and SVR. Referring to the parameter setting of similar problems in the existing literature, different parameters are set to conduct the Taguchi experiments. According to the experiment results, when the population size is 10 and the number of iterations is 25, the satisfied results can be obtained with appropriate running time. In SVR model, the training set scale is 0.7, the remaining is testing set. The range of parameter *C* and *g* are both taken (0,100). The following Fig. 4 is the fitness variation curve of parameter optimization.

**Figure 5:**
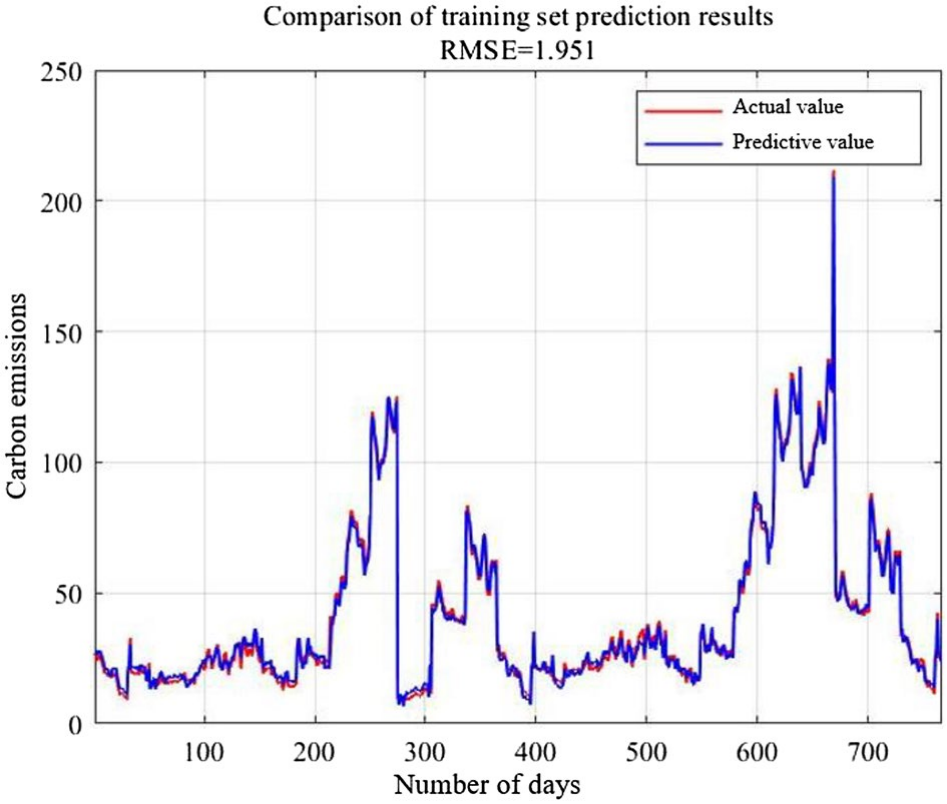
Prediction comparison of training set.

**Figure 6:**
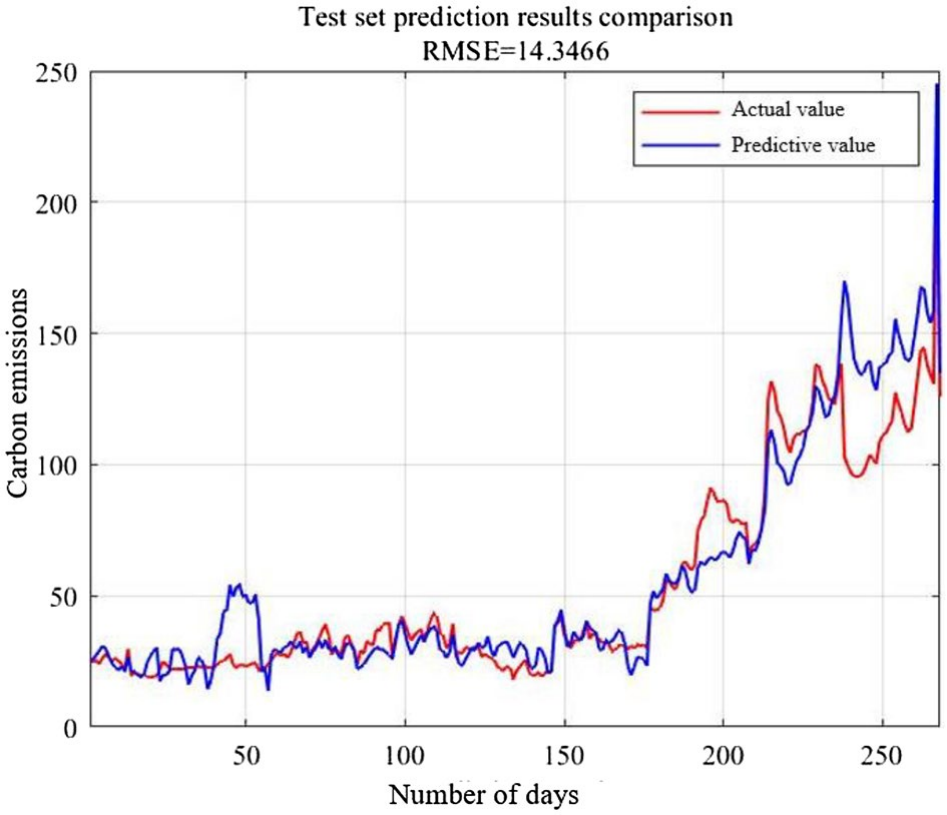
Prediction comparison of testing set.

The optimal penalty parameters *C* and *g* are obtained as 22.6580 and 8.9441 respectively, which is optimized by GA. Analyze the prediction results of the GA–SVR model with the training set and the testing set, the comparison curves are shown in Figs. 5 and 6.

From the two comparison curves of Figs. 5 and 6, the fitting accuracy of the raining set prediction results by GA–SVR performs excellent. The curve characteristics perform basically consistent. The RMSE is 1.951 merely, while the RMSE of testing set is 14.3466. The R^2^ of the training set achieves 0.9960, while the R^2^ of testing set exceeds 0.85. Although testing set prediction curve deviates from the expected value, the curve change trend can reflect the change of the true value.

### Prediction results analysis of compared algorithms

In order to verify the validity of the prediction model in this paper, a SVR with random parameters (C = 4, g = 0.8), the BP neural network (BP) and Random Forest (RF) models are introduced to compare with GA–SVR. Three metrics R^2^, MAE and MBE are utilized to evaluate the prediction effect of the models. The results are as follows.

In Table 1, the improved GA–SVR model performs excellent on the carbon emissions prediction of electricity consumption in urban communities. The fit correlation coefficients between prediction values and actual values achieves 0.9960 and 0.8601 respectively for the training set and the testing set. They both reaches more than 85%. The community electricity carbon emission forecast is accurate. The training and testing set of the improved algorithm fit more accurately than the other three comparison algorithms.

**Table 1:** Comparison of prediction algorithms.

Model	Training set			Testing set		
	R^2^	MAE	MBE	R^2^	MAE	MBE
GA–SVR	0.9960	1.7800	− 0.0742	0.8601	9.2341	2.9112
SVR	0.6974	9.9612	− 1.2350	0.7284	13.9492	− 6.9973
BP	0.5412	14.1563	− 2.5174	0.5410	15.8081	− 11.9257
RF	0.9328	4.7816	0.0648	0.7073	13.8018	− 7.3491

For the stability comparison, the four comparison algorithms, GA_SVR, SVR, BP and RF, are independently run for 20 times. The evaluation indicators are obtained as shown in Table 2. The horizontal direction represents the worst values (min), the best values (max), the average values (mean) and the standard deviations (std) of the prediction accuracy R^2^ of the training set and the test set.

**Table 2:** Comparison index performance of R^2^.

Model	Comparison index of training set R^2^				Comparison index of testing set R^2^			
	Min	Max	Mean	Std	Min	Max	Mean	Std
GA–SVR	**0.9882**	**0.9963**	**0.9916**	0.0027	**0.8235**	**0.8661**	**0.8488**	0.0115
SVR	0.6958	0.6958	0.6958	**0.0000**	0.6899	0.6899	0.6899	**0.0000**
BP	0.3468	0.7106	0.5483	0.0967	0.3627	0.6050	0.5157	0.0631
RF	0.9238	0.9325	0.9283	0.0023	0.6655	0.6982	0.6882	0.0072

The optimal values of each evaluation index are in [bold].

It can be seen from the statistical results in Table 2 that GA_SVR algorithm performs best in the solution process. When the determined parameters of SVR, the solution is the most stable, but the result metrics are not very well. The GA_SVR solution process performs both well and stable, while GA is introduced to improve the parameters of SVR. The optimal value, the worst value and the average value perform more outstanding than other compared algorithms.

Since electricity consumption data may be affected by uncertainties such as the epidemic, the carbon emissions of community electricity consumption have shown a significant upward trend in a certain period. So, the data uncertainty is strong. After the improvement, the fitting accuracy is greatly improved compared with the random parameter SVR.

### Future carbon emissions forecast

After substituting the annual emission factor curve of the power system from 2019 to 2021 and the average daily electricity consumption of residents into the trained GA–SVR model. The trend comparison between the actual and predicted carbon emissions (tCO_2_e) of the community in January 2022 is obtained, and the prediction accuracy R^2^ is 84.33%.

**Figure 7:**
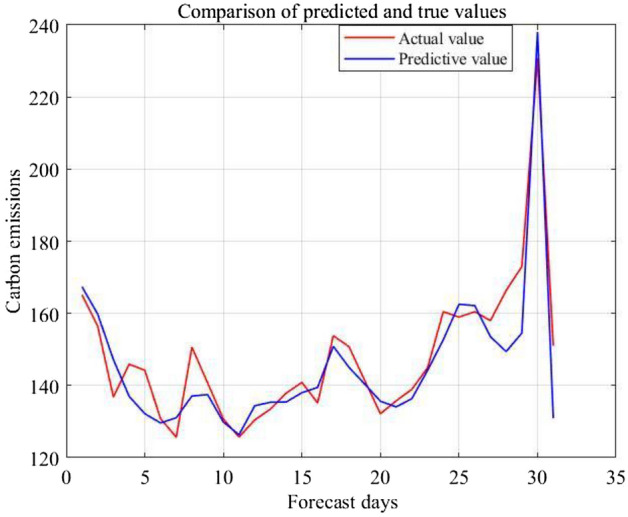
Carbon emission forecast and actual values for January 2022.

As shown in Fig. 7, the prediction results are consistent in the actual carbon emission data of electric power. In the Caochang Community Energy Big Data Management System, the electricity consumption of the community can be calculated in real time. After the prediction model is modified according to the latest data, the carbon emission data in the next 30 days can be estimated with a high accuracy. It performs guiding significance for community carbon emission early warning within 1 month.

### Carbon emission early warning mechanism and suggestions for community residents emission reduction

#### Design of carbon emission early warning mechanism

As the implementation of the replacing coal with electricity policy, the electricity carbon emissions increase for a period, which is mainly due to the increase in electricity consumption. However, with the stabilization of electricity consumption and the control of carbon emissions from power supplies, the effect of carbon emissions reduction will become more and more significant.

According to the carbon emission prediction results of community residents, the energy consumption analysis, energy saving and efficiency, and convenient power connection will be provided. If the forecast carbon emissions exceed a certain threshold compared with the same period of previous years, the previous month and the previous day, a carbon emission early warning will be issued to remind residents^35^.

**Figure 8:**
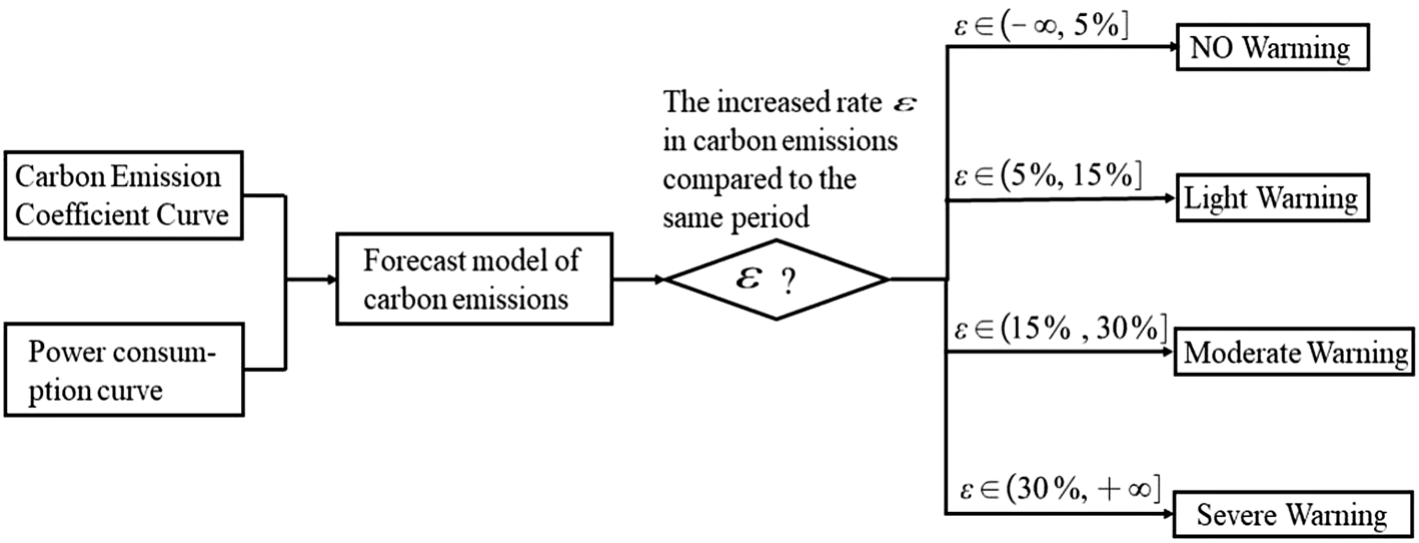
Community carbon emission warning process.

As shown in the Fig. 8, carbon emissions in 1 month can be predicted. If the forecast monthly carbon emissions and the forecast results of a certain day exceed the threshold of the early warning, carbon emission warning will be issued to residents of the community and the rational use of electricity will be reminded.

#### Recommendations of community carbon reduction strategy

Northern communities prefer to keep warm with coal. With the implementation of gas to electricity, coal to electricity policy, community electricity carbon emission has grown significantly. On the premise of carbon emission reduction of NCPG power supply, the community will also achieve carbon emission reduction from the following perspectives:

New energy as a power supply has a lot of improvement space. In the process of building a new power system, solar, wind and other new energy power generation will gradually become the main power source. For example, the construction of intelligent micro-network parking lot, the exploration of variable charging integrated box-type transformer, DC V2G charging pile and other technical applications. All these will provide basic guarantee for regional green transportation and facilitate resident travel.

Implement intelligent transportation strategy. Select Xixinglong Street for street lamp reconstruction and layout intelligent control street lamps. Install photovoltaic panel lampshade, intelligent control device, wireless charging, WIFI, video surveillance, information announcement screen and other value-added functions. Realize intelligent lighting of street lamps, self-use, complementary control of municipal electricity, and promote the parallel of intelligent transportation and green transportation. To solve the problem of charging electric bicycles for common people, two centralized charging sheds will be selected in North Lucao Garden and Xixinglong Street for photovoltaic transformation. Provide convenient, safe and green charging experience for community residents, and guide residents to change their travel mode.

Strengthen the promotion of green and low-carbon life. Improve community residents' awareness of low carbon environmental protection from various aspects, such as food, clothing, housing and transportation, which will promote carbon emission reduction in life.

## Conclusion

This paper calculates the emission coefficient of the power system, and constructs a carbon emission fitting curve. Based on the community electricity consumption data and the dynamic carbon emission coefficient, a rapid carbon emission prediction model is constructed and trained. Although the carbon emission prediction has achieved ideal results, the research in this paper hasn’t take into account the national policies and the key factors that affect the carbon emission change of electric energy. There is still much room to optimize the results of the testing sets. In future, the above factors would be considered for further research.

## Supplementary Information

Supplementary Information 1.

Supplementary Information 2.

## Data Availability

All data generated or analyzed during this study are included in supplementary file Code&data.
