# Supplementary material for: Research on the community electric carbon emission prediction considering the dynamic emission coefficient of power system
Source: Sci Rep. 2023 Apr 5;13:5568. doi: 10.1038/s41598-023-31022-y (PMC10076421; doi:10.1038/s41598-023-31022-y)
Supplement: Supplementary file 2 — Supplementary Information 2. [file 41598_2023_31022_MOESM2_ESM.docx]

Description of code and data

The files in the attachment are the code of the four algorithms for the experiment, as well as the data required to run the program.

The following is a detailed description of the four algorithms.

Data File

| supplementary table S1.xlsx | Carbon emission data |
| --- | --- |

Description: This table is the calculated daily carbon emissions of the community and used for the training of carbon emission prediction model. When each algorithm runs, the file needs to be placed into the corresponding folder.

GA-SVR

| Program file name | Instruction |
| --- | --- |
| main.m | Main function |
| bs2rv.m | This function decodes binary chromosomes into vectors of reals. |
| crtbase.m | This function creates a vector containing the base of the loci in a chromosome. |
| crtbp.m | This function creates a binary population of given size and structure. |
| gaSVMcgForRegress.m | Genetic algorithm to optimize the parameter code |
| libsvm Parameter Description.txt | This section describes SVR algorithm parameters |
| mut.m | This function takes the representation of the current population, mutates each element with given probability and returns the resulting population. |
| ranking.m | This function performs ranking of individuals. |
| recombin.m | This function performs recombination between pairs of individuals and returns the new individuals after mating. The function handles multiple populations and calls the low-level recombination function for the actual recombination process. |
| reins.m | This function reinserts offspring in the population. |
| rep.m | This function replicates a matrix in both dimensions. |
| select.m | This function performs universal selection. The function handles multiple populations and calls the low level selection function for the actual selection process. |
| sus.m | This function performs selection with Stochastic Universal Sampling. |
| svmpredict.mexw64 | Svr predict code |
| svmtrain.mexw64 | Svr train code |
| xovmp.m | This function takes a matrix OldChrom containing the binary representation of the individuals in the current population, applies crossover to consecutive pairs of individuals with probability Px and returns the resulting population. |
| xovsp.m | This function performs single-point crossover between pairs of individuals and returns the current generation after mating. |

The operating environment is "Matlab2020". Place ‘supplementary table S1.xlsx’ in GA-SVR folder, double click to run ‘main.m’.

SVR

| Program file name | instruction |
| --- | --- |
| libsvm Parameter Description.txt | This section describes SVR algorithm parameters |
| main.m | Main function |
| supplementary table S1.xlsx | Carbon emission data |
| sus.m | This function performs selection with STOCHASTIC UNIVERSAL SAMPLING. |
| svmpredict.mexw64 | Svr predict code |
| svmtrain.mexw64 | Svr train code |
| svmpredict.mexw64_1511296.bak | Svr prediction toolbox |
| svmtrain.mexw64_1511296.bak | Svr train toolbox |

The operating environment is "Matlab2020", Place ‘supplementary table S1.xlsx’ in SVR folder, double click to run ‘main.m’.

RF

| Program file name | instruction |
| --- | --- |
| main.m | Main function |
| supplementary table S1.xlsx | Carbon emission data |

The operating environment is "Matlab2020", Place ‘supplementary table S1.xlsx’ in RF folder, double click to run ‘main.m’.

BP

| Program file name | instruction |
| --- | --- |
| main.m | Main function |
| supplementary table S1.xlsx | Carbon emission data |

The operating environment is "Matlab2020", Place ‘supplementary table S1.xlsx’ in BP folder, double click to run ‘main.m’.
